# Music, autism, and emotion

**DOI:** 10.3389/fpsyg.2013.00890

**Published:** 2013-12-06

**Authors:** Nick Zangwill

**Affiliations:** Philosophy Department, Hull UniversityHull, UK

**Keywords:** music, autism, emotion, experience, listening strategy

I argue that musical experience has little to do with understanding or imagining emotion. I draw on experimental work done by Rory Allen and his colleagues on musical experience and autism.

## Autism

In order to pursue the argument, we need some general and relatively uncontroversial claims about autism and emotion. For brevity, by people with “autism,” I will mean people with what is termed “autism spectrum disorder”—which encompasses a variety and a range of different conditions, but which all involve a deficiency in thinking about other minds. Two decades ago there was a consensus that the explanation was that those with autism were to some extent “mind-blind”; that is, those with autism exhibit a relative lack of understanding of other minds when compared with typically developing people (Baron-Cohen, 1995). But this is not so widely accepted now. Other possible explanations have been suggested. For example, a rival hypothesis is that other-mind thought creates stress, which inhibits thinking in those terms (see Rieffe et al., 2000). On this view there is no cognitive shortfall as there is on the mind-blindness hypothesis; instead other-mind thinking is disrupted by stress. It has even been suggested that autism is not a unitary condition, in which case there is no single explanation to be had (Happe et al., 2006). Nevertheless, whatever the explanation, those with autism systematically do worse in similar circumstances in *attributing* emotion states to other minds. Few doubt that those with autism have a problem with attributing mental states to others. *That* remains relatively uncontroversial and a consensus view. [There are dissenting voices: see for example Tracy et al. (2011). But the results cited in that paper only concern a highly structured task and are not generalizeable. Compare Allen et al. (2013).]

Despite divergences, most current theories of autism agree that autistic people are less well-functioning, not in their *possession* of emotions but (A) in the ability to *attribute* emotions to others, (B) in the ability to *imagine* emotions when not having them, and (C) in their ability to *describe* emotions in language. On this last point, 85% of those who are independently identified as having autism have what is called “Alexithymia II,” which is a shortfall in the ability to name their own emotions (Hill et al., 2004). Alexithymia II indicates a defect in understanding one's own emotions. These shortfalls have been probed and the difference between autistic groups and non-autistic groups in ascribing, imagining and describing emotion is statistically significant, and the results replicated [Hobson, 1993; Frith, 2003; see also Bird et al. (2010) for the view that the defect in self-knowledge explains defects in thinking about other minds]. There are a variety of theories of what autism is, what explains it, and of exactly what abilities such a person has or lacks. But most psychologists accept that in general and in similar circumstances, the psychological ascriptions by those with autism are less accurate than those of typically developing people, and that, whatever the ultimate explanation, thinking in terms of the mental states of others is more difficult for those with autism than for typically developing people. This much remains widely accepted.

Part of the shortfall of autistic people with respect to the emotions of others is that they find it hard to ascribe emotions that they do not feel. That is, there is a shortfall not only in respect of knowledge but also in respect of imagination. The well-known false-belief puppet tests show that autistic people do worse at conceiving of others as having beliefs and other mental states that they themselves lack (Baron-Cohen, 1995). And this derives from differences in the ability to imagine mental states they lack. The interpretation of our general ability to think about other minds is controversial (there was once a “simulation vs. theory-theory” debate). But all sides agree that understanding other minds implies being able to conceive or imagine other people having states that the ascriber lacks. (Imagination in this sense does not necessarily mean imagining what is not the case; we may imagine what is now going on in New Zealand.)

## Musical experience and autism

With these general remarks about autism on the table, let us now turn to music. One question is: what is the autistic person's experience of music like? Perhaps a better question is a comparative one: how, if at all, do the autistic and non-autistic person's experiences of music differ? We need a relatively neutral empirical investigation of this. We need data comparing autistic and non-autistic musical experience.

The use of questionnaires is obviously inappropriate for studying the musical experiences of autistic people. For they are less able than non-autistic people in applying *linguistic* emotion descriptions to themselves (Alexithymia II). We need access to musical experience, apart from self-reports.

One way forward is to use *physiological* responses to music as evidence. This is what Rory Allen and his collaborators have pursued in a number of papers. He used galvanic skin responses (“GSR”), which are generally accepted as a non-verbal measure of general physiological arousal, as a non-verbal measure of people's response to music (Allen et al., 2013). “Physiological arousal,” in this sense, does not necessarily indicate ordinary (non-musical) emotions of the anger, grief, pride variety. Many ordinary emotions involve physiological arousal, and they are accompanied by GSR, but there can be physiological arousal without ordinary emotions. That is, ordinary emotions generate GSR but GSR does not necessarily indicate ordinary emotions.

The suggestion is that we may take the GSR tests to measure what we may call “musical experience,” in a general and neutral sense. This is an extra assumption. But we may accept it, given the particular experimental context in which GSR occurred. In the experimental situation the response occurred given the music prompt and not without it. Random non-musical noise did not produce the response. So this makes it very likely that the response is *to* the music, in the sense that not only is it caused by the music but it indicates a psychological state that is about the music—the content of which is the music. Furthermore, although GSR arousal can reflect positive or negative arousal, there is no reason not to assume the responses to be a positive rather than negative—although for the argument of this paper, it does not matter whether the response is positive or negative so long as it indicates musical experience. GSR signals musical experience and the lack of GSR (in those experimental circumstances) signals lack of musical experience. Saying that musical experience is indicated by GSR is neutral in itself about the nature of those experience, which is exactly why they can be used as part of an argument for their nature.

Hence, other things being equal, in most cases, and in the experimental situation in which musical stimulus was provided, GSR indicates musical experience, which in most cases can be assumed to be a positive felt response of some kind or other. The question is: what kind?

## The results and the argument to musical experience in general

Allen and associates compared the physiological responses of autistic and non-autistic musical listeners. Participants in the experiment listened to a standard set of musical stimuli, and their physiological reactions were compared with a control set of environmental noise stimuli, so as to filter out responses to sound as such, rather than to music. (The music was of a sort familiar to both groups of listeners.)

What were the results?

What Allen and associates found was that autistic listeners do respond physiologically to music, and—and this is the very interesting result—they respond to a similar degree to typical non-autistic listeners. That is, the result was: there was *no* significant difference between autistic and non-autistic control groups. They wrote:

“The results indicated that there was no sign of any reduced responsiveness to the musical stimuli, at this physiological level, in the ASC group compared with the control group.” (Allen et al., 2013, p. 440.)

Autistic listener's felt responses to music is (statistically) normal. Of course there are a diversity of autism phenomena. But in what are called “high-functioning” cases of autism, the musical response, as measured by GSR, was not significantly different from the non-autistic control group. [For some similar results see also Heaton et al. (2001) and Heaton (2009). Khalfa and Peretz (2007) report enhanced GSR responsiveness in autistic listeners; if that were correct it would strengthen the argument.]

Now what do these results about physiological arousal result tell us? Physiological arousal is indicative of music experience, *whatever it is*. Music experience might be a variety of kinds of mental states, such as sensation, emotion, imagination, belief. It does not matter for our purposes. What is important is that physiological arousal is consequential on music experience, whatever it is, and the interesting fact is that it is statistically normal for autistic listeners.

The GSR response, in itself, is neutral concerning the nature of the musical response (the state of mind of musical experience)—whether it is an ordinary emotion, such as grief, pride or anger, or the idea that it is a specifically musical emotion, or the idea that it is a pleasure in the beauty of the music. GSR would measure *all* this.

However, the GSR results, when *combined* with general facts about autism, give us a strong argument, which is this:

1. Autistic and non-autistic groups differ with respect to understanding and imagining emotion.
2. But they do not differ in the physiological arousal that is indicative of musical experience.
3. So whatever the musical experience is, it should not be understood in terms of understanding or imagining emotions.
4. If it were, autistic and non-autistic listeners would be significantly different. But the GSR results show that they are not.

There are merely logically possible hypotheses that this argument ignores. One is that music uniquely triggers normal emotion cognition in autistic listeners. But there is too much independent evidence for a general capacity shortfall to make musical listening somehow an exceptional miraculous cure for autism. Autistic people have emotions but the independent evidence for Alexithymia II) among most of those with autism is strong. Another logically possible idea would be that autistic listeners are indeed responding positively and they are listening to the same sounds, but they are somehow listening to the sound but not as music. However, the noise control ruled out the response being to noise. Are they responding only to rhythm, say? There is no more reason to think this than there is to think that red-haired people listen to music differently. Given that we do not have to worry about such bare possibilities, we can make a strong inference to the best explanation to the effect that the musical experiences of both autistic and control group listeners involves neither understanding nor imagining emotion.

Two comments:

- The position defended here fits neatly with the prevalence and effectiveness of music therapy on people with autism. It is effective with autistic people precisely because they do not have to think in terms of psychological states. That is why music works well with them. (I gather that something parallel is true of visual designs and rhythms, as opposed to representations of people expressing psychological states.)
- The view also fits neatly with the fact that there are gifted autistic musicians. Presumably musical experience is a constituent part of what is necessary for musical performance. If so, according to the emotion theories of music that I am targeting, there should be no gifted autistic musicians. But there are. (These musicians tend to be soloists rather than orchestral and chamber group players Miller, 1989).

## Coda

The argument is this. Physiological responses show that the music experiences of autistic people are normal in comparison with the musical experiences of non-autistic people. But their emotion understanding, imagination and description is not. Therefore, *both* autistic and non-autistic musical experience is independent of their emotion understanding, imagination, and description.
